# Diminished hedonic capacity in social activities as a mediator of the link between dysfunctional behavioral activation system and depressive symptoms

**DOI:** 10.3389/fpsyt.2024.1337847

**Published:** 2024-02-06

**Authors:** Chi-son Kuan, Qian-yu Liu, Gang-min Xu, Han-yu Zhou, Jia-hui Nie, Chao Yan

**Affiliations:** ^1^ Shanghai Changning Mental Health Center, Shanghai, China; ^2^ Key Laboratory of Brain Functional Genomics (MOE&STCSM), Affiliated Mental Health Center (ECNU), School of Psychology and Cognitive Science, East China Normal University, Shanghai, China; ^3^ Shanghai Key Laboratory of Mental Health and Psychological Crisis Intervention, School of Psychology and Cognitive Science, East China Normal University, Shanghai, China

**Keywords:** behavioral activation system, social anhedonia, hedonic capacity, depressive symptoms, adolescent

## Abstract

**Background:**

Adolescence is a crucial period for the development of depression, and previous studies have suggested that the Behavioral Activation System (BAS) plays a significant role. However, little is known about the underlying mechanisms. This study aimed to explore the mediating role of anhedonia in the relationship between BAS and depressive symptoms among Chinese adolescents.

**Method:**

A total of 1,023 high-school students aged 15–18 years participated in the study, with 916 continuing their participation three months later. All participants completed the Behavioral Inhibition System/Activation System (BIS/BAS) scale, Dimensional Anhedonia Rating Scale (DARS), Children’s Depression Inventory (CDI), and the State-Trait Anxiety Inventory (STAI-S/T). Pathway model analysis was performed to examine the concurrent and prospective mediating effects of anhedonia and the potential moderating effect of sex.

**Result:**

Anhedonia in the domains of social activities, hobbies and sensory experiences significantly mediated the cross-sectional relationship between BAS and depressive level three months later. Furthermore, the beta-value of the mediating effect of social activities was significantly higher than that of the other domains of hedonic capacity cross-sectionally and longitudinally. However, sex showed no significant moderating effect.

**Conclusion:**

Our findings underscore the importance of hedonic capacity, especially within the social domain, in the development of depressive symptoms. These findings contribute to the early diagnosis and prevention of depressive disorders.

## 1 Introduction

Childhood and adolescence represent periods of high risk for the development of depression. In China, the prevalence of depressive symptoms among Chinese adolescents reached 24.9% in 2020 (1),with depressive adolescents accounting for 30.28% of clinical depressive group in China (2). Depression that develops during adolescence is likely to persist into adulthood (3), with high suicide and disability rates, and severe negative effects on adolescents’ physical and mental health (4).

Drawing upon Reinforcement Sensitivity Theory, the behavioral activation system (BAS) corresponded to reinforcing events and behaviors (5). BAS is involved in appetitive/approach motivation and is sensitive to signals of “reward and punishment avoidance”. The diminished sensitivity of the BAS has been linked to a higher incidence of affective disorders (6, 7). Additionally, alterations in BAS have shown a significant correlation with the response to antidepressant treatment. Notably, the effectiveness of behavioral activation therapy (BAT) can be elucidated through BAS, as BAT’s core mechanism involves the ‘activation’ of motivational behavior (8) and the reduction of ‘avoidance’ behavior (9–11). Neuroscience research has shed light on the sensitivity of dopaminergic reward system and prefrontal cortex towards frustrated experience mediated between the decrease of approach motivation and depression (12). However, conclusive evidence supporting the hypothesis that abnormal BAS levels directly lead to difficulties in reward processing, such as anhedonia, which ultimately contributes to the manifestation of depressive symptoms, is currently lacking.

Anhedonia, defined as the loss of interest and pleasure from previously enjoyed experiences (13). It has been reported that anhedonia level was negatively correlated with BAS level among healthy participants (14). Similarly, early positive changes in the levels of BAS components (i.e., increased reward responsiveness level in the first 2 weeks) might also predict the improvement of anhedonia performance 6 weeks later in patients with depression under medical treatment (15). Besides its association with motivation systems (particularly BAS), anhedonia also played an important role in the development of depression. Individuals with “fear of positive evaluation”(i.e., feeling afraid or worrying from receiving positive evaluation publicly) and anxiety might develop depressive symptoms through the longitudinal mediating effect of anhedonia (16, 17). Moreover, the emergence of anhedonia in adolescence might reflect the later development of depression (18).

Studies on anhedonia have attempted to variations in domains of hedonic capacity across various contexts (such as hobbies, food/drinks, social activities, and sensory experiences) (19). During adolescence, defects in experiencing pleasure, particularly from social interactions, might produce more negative effects on psychosocial development and functioning (20, 21) as sensitivity to social and cultural signals increases during this key period (22). Defects in experiencing social pleasure, avoidance of social relationships and situations may lead to a lack of social emotional support, limited opportunities for social-cognitive growth and developing social skills, and persistent loneness in adulthood (23). Hence, it is probable that anhedonia in the social domain functions serves as the most robust mediator in both cross-sectional and longitudinal analyses, alongside other domains. Although the mediating role of anhedonia has been discussed in previous study (24), the measurement and comparison of anhedonia within different domains as mediators have not been examined among healthy adolescents.

Additionally, it is noteworthy to consider the gender gap and explore sex differences in terms of prevalence and vulnerability (25). During adolescence, females tend to report greater depressive symptoms than males (26) because of bodily factors (e.g., significant change of hormone) and social factors (e.g., ruminative coping strategies) (27). Therefore, the role of sex in BAS, anhedonia, and depression remains heavily debated.

In this study, we aimed to investigate and compare the mediating role of different domains of anhedonia in the relationship between BAS and depressive symptoms. Moreover, we examined the moderating role of sex in a model involving BAS and depressive symptoms. Accordingly, three hypotheses were proposed. First, BAS levels can predict a decrease in anhedonia levels, which further predicts a lower level of depressive symptoms cross-sectionally and longitudinally. Second, the social activities domain of anhedonia compared with other domains measured in this study (i.e., hobbies, food/drink, and sensory experience) would uniquely show the most stable and strongest indirect effect on the relationship between BAS and depressive symptoms. Finally, sex would moderate the relationship between BAS and depressive symptoms and between BAS and anhedonia.

## 2 Methods

### 2.1 Participants and procedure

Participants were recruited from a randomly selected senior high school in a city in western China. All students were invited to participate in the initial survey through flyers and posters. Ultimately, a total of 1,035 students completed the first-round survey. After excluding 12 subjects who reported psychiatric disorders, a family history of mental illness, and/or were older than 18 years old, the data of 1,023 students were included in the analysis (valid rate: 98.84%) and 916 students agreed to continue in the follow-up survey 3-months later (dropout rate: 10.46%). The participants had a mean age of 17.02 ± 0.76 (range, 15–18) years during the initial survey; more than half of the participants were females (61.5%). All participants provided written informed consent in the first-wave survey. This study was approved by the Ethics Committee on Human Research of East China Normal University (HR 257-2021).

### 2.2 Measurements

#### 2.2.1 The behavioral inhibition system/behavioral activation system scale

The BIS/BAS scale was used to assess the participants’ sensitivity to avoidance motivation and approach motivation (28). The original BIS/BAS scale included 24 items, evaluated using a 4-point Likert scale (1 = *strongly agree*, 2 = *agree*, 3 = *disagree*, 4 = *strongly disagree*). In this study, lower scores indicated stronger BIS and BAS sensitivity. The BAS subscale (17 items) can be further divided into the following: BAS-Drive (BAS-D), BAS-Fun Seeking (BAS-F), and BAS-Reward Responsiveness (BAS-R). The BAS-D scale includes items related to the continuous pursuit of a desired goal. The BAS-F scale includes items that correspond to seeking new rewards and the excitement of the rewarding moment. The BAS-R includes items representing the anticipation or occurrence of a reward. Low BIS scores indicate stronger motivation to avoid aversive or punishing stimuli; high BIS sensitivity might be related to anxiety and depression (5). In contrast, lower BAS scores represent a stronger level of motivation to approach rewarding stimuli, and BAS sensitivity has been reported to be negatively related to depression. The Chinese version of BIS/BAS scale has demonstrated acceptable internal consistency (Cronbach’s *α* = .79), test–retest reliability (*r* = .80), and construct validity (the root mean square error of approximation (RMSEA) = .06, the goodness of fit index (GFI) = .93, the average goodness of fit index (AGFI) = .91, the Incremental Fit Index (IFI) = .86, the comparative fit index (CFI) = .86, the Tucker Lewis index (TLI) = .84) among students aged 12–24 years (29). In this study, the intraclass correlation coefficient (ICC) for calculating the test–retest reliability of the BIS and BAS was.65 and.64, respectively. Cronbach’s α for the BIS/BAS Scale was.86, and that for its subscales was.45–.81.

#### 2.2.2 The dimensional anhedonia rating scale

The DARS, which comprises 17 items, was employed to measure the levels of anhedonia symptoms (19). This scale has four subscales, including hobbies, food/drink, social activities, and sensory experiences. Each subscale begins with a fill-in-the-blank question where the participants must list at least two things/experiences within a specific scenario. Then, the participants were required to answer four or five questions (e.g., “I would enjoy these activities” and “I would actively participate in these social activities”) with rating using a five-point Likert scale (i.e., 0 = not at all, 1 = slightly, 2 = moderately, 3 = mostly, 4 = very much). A lower score indicates a higher level of state anhedonia. The Chinese version of the DARS yielded good validity (model fitness index: *χ2/df* = 2.02, *GFI* = .88, *RMSEA* = .08, *CFI* = .96, *TLI* = .96) and reliability (Cronbach’s α = .97 for the entire scale and.89 –.96 for the subscales) (30). In this study, regarding test–retest reliability, the ICC for was .60 for the hobbies domain subscale (*p* <.01), .63 for the food/drink subscale (*p* <.01), .70 for the social activities subscale (*p* <.01), and .64 for the sensory experiences subscale (*p* <.01). Cronbach’s *α* was .90 for the hobbies domain subscale, .78 for the food/drink subscale, .89 for the social activities subscale, and .90 for the sensory experiences subscale.

#### 2.2.3 Children’s depression inventory

The CDI was used to measure the level of depression in children and adolescents aged 7–17 years (31). This tool has 27 items encompassing five subscales (i.e., anhedonia, negative mood, negative self-esteem, ineffectiveness, and interpersonal problem). Each item contains three choices describing the severity of depressive symptoms (0–2 scores). A higher total score indicates more severe depressive symptoms. The Chinese version of the CDI shows good internal consistency (*Cronbach’s α* = .88), test–retest reliability (*r* = .81), and construct validity (*χ2 =* 1504.65, *df* = 314, *χ2/df* = 4.79, *TLI* = .88, *NNFI* = .87, *RMSEA* = .038) (32). In this study, the test–retest reliability calculated using ICC was .86 (*p* <.01), and the internal consistency Cronbach’s *α* was .86.

#### 2.2.4 State-trait anxiety inventory

The STAI, which includes two subscales of state anxiety (STAI-S) and trait anxiety (STAI-T), was employed to measure anxiety levels (33). Both subscales consist of 20 items, each of which is scored using a four-point Likert scale, ranging from 1 to 4. A higher score indicates a greater level of anxiety. Both the STAI-S and STAI-T subscales showed good internal consistency with Cronbach’s α of.89 and construct validity for the entire scale (*χ2 =* 1571.328, *df* = 169, *p* <.001, *CFI* = .911, *TLI* = .900, *RMESA* = .061, *SRMR* = .039) among healthy Chinese university students (34). The test–retest reliability calculated using the ICC of the STAI-S and STAI-T subscales was .78 and .80, respectively. The internal consistency Cronbach’s *α* was .91 and .78 for the STAI-S and STAI-T subscales, respectively.

### 2.3 Data analysis

IBM SPSS Statistics for Mac, Version 22.0 was used to generate descriptive statistics. Missing values were replaced by the mean of the target item for the total sample. The Pearson correlation was used to calculate the relationship among the major variables, including the sensitivity of avoidance and approach motivation, anhedonia, level of depressive symptoms, and state/trait anxiety. Previous research has suggested that the likelihood of false positive results increases with the number of tests conducted. When the number of hypothesis tests exceeds 100, the family-wise error rate (FWER; probability of at least one Type I error among multiple tests) exceeds 99% (35). Given that a total of 153 correlation tests were conducted between variables, it is crucial to control the Type I error to decrease the risk of false-positive findings. The Bonferroni correction is a method that mitigates the risk of Type I error in multiple hypothesis tests by adjusting the significance level. The statistical significance of correlation tests was determined using the Bonferroni-adjusted p-value, calculated as recommended by Machin (36), at 0.05 divided by the number of tests performed in correlation analysis (0.05/153 = 0.000327). Independent sample *t*-tests were also performed to evaluate differences in the main variables between the two sex groups.

Structural equation models were constructed to assess cross-sectional and longitudinal mediation models of BAS, domains of anhedonia, and depressive symptoms. The mediating effects of the four domains of anhedonia, including hobbies, food/drink, social activities, and sensory experiences, were examined in four models cross-sectionally and longitudinally. State anxiety and trait anxiety were entered as covariates for anhedonia and depressive symptoms because anxiety might predict depression through the indirect effect of anhedonia (17, 37). Eight cross-sectional mediation models were constructed using the data collected from two waves. Additionally, four longitudinal mediation models were proposed. Within the longitudinal model, all four domains of anhedonia at Wave 2 were included as mediators, whereas the CDI score at Wave 2 was designated as the outcome variable. To account for potential influences, state anxiety and trait anxiety at Wave 2 were included as covariates in the analysis. Furthermore, moderated mediation models were constructed based on the cross-sectional and longitudinal mediation models to assess the moderating effect of sex on the mediating role of anhedonia between BAS and depressive symptoms. In Mplus, bootstrap confidence intervals (CI) were employed to determine the significance of indirect effects. A bias-corrected bootstrapping technique with 10,000 resamples was used to obtain a 95% CI. A CI that does not include 0 indicates a significant model fit.

## 3 Results

### 3.1 Descriptive statistics and bivariate relationships

The descriptive statistics of the main variables, together with the results of the correlation test, are shown in **Table 1**. Even after employing the Bonferroni correction for both baseline and longitudinal data, BAS scores remained positively correlated with CDI scores, suggesting an inverse relationship between depression levels and behavioral activation sensitivity. In addition, only BAS scores were significantly correlated with DARS scores cross-sectionally and longitudinally. Because lower DARS scores indicated a higher anhedonia level, the relationship between BAS and DARS subscales suggesting higher sensitivity of behavioral activation was associated with lower anhedonia levels. BIS scores were negatively correlated with CDI scores, indicating a positive relationship between depressive symptoms level and behavioral inhibition sensitivity. However, the BIS scores did not show any significant correlation with the subscales of the DARS at Wave 1 and Wave 2 after applying the Bonferroni correction.

**Table 1:** Summary of descriptive characteristics and bivariate correlations among all the relevant variables.

Variable	*n*	*M*	*SD*	1	2	3	4	5	6	7	8	9	10	11	12	13	14	15	16	17	18
1. BIS (W1)	1023	8.3	2.79	-																	
2. BAS (W1)	1023	24.55	6.07	.38*	-																
3. DARS - H (W1)	1023	13.16	3.07	-.06	-.21*	-															
4. DARS - FD (W1)	1023	11.09	3.35	-.08	-.24*	.34*	-														
5. DARS – SA (W1)	1023	11.96	3.48	.06	-.24*	.33*	.38*	-													
6. DARS – SE (W1)	1023	15.59	4.08	-.05	-.27*	.46*	.45*	.45*	-												
7. CDI (W1)	1023	16.14	7.44	-.32*	.21*	-.14*	-.07	-.36*	-.17*	-											
8. STAI-S (W1)	1023	44.91	10.96	-.33*	.14*	-.21*	-.08	-.33*	-.20*	.69*	-										
9. STAI-T (W1)	1023	47.35	8.59	-.39*	.13*	-.14*	-.06	-.30*	-.16*	.73*	.83*	-									
10. BIS (W2)	916	8.61	3.06	.49*	.13*	-.12*	-.08	.08	-.05	-.27*	-.20*	-.27*	-								
11. BAS (W2)	916	24.91	6.57	.13*	.47*	-.20*	-.19*	-.17*	-.23*	.12*	.12*	.12	.51*	-							
12. DARS – H (W2)	916	13.05	3.31	-.11	-.20*	.42*	.23*	.17*	.25*	-.16*	-.17*	-.10	-.09	-.22*	-						
13. DARS – FD (W2)	916	11.19	3.32	-.08	-.21*	.28*	.47*	.25*	.31*	-.11	-.14*	-.11	-.03	-.25*	.47*	-					
14. DARS – SA (W2)	916	11.89	3.65	-.01	-.23*	.28*	.25*	.55*	.36*	-.32*	-.29*	-.26*	.05	-.24*	.43*	.46*	-				
15. DARS – SE (W2)	916	15.42	4.17	-.05	-.26*	.33*	.29*	.34*	.48*	-.22*	-.19*	-.17*	0	-.28*	.47*	.55*	.57*	-			
16. CDI (W2)	916	16.45	7.83	-.23*	.13*	-.11	-.01	-.30*	-.14*	.75*	.58*	.63*	-.25*	.19*	-.25*	-.17*	-.40*	-.28*	-		
17. STAI-S (W2)	916	45.34	11.02	-.23*	.10	-.15*	-.03	-.26*	-.13*	.58*	.64*	.59*	-.22*	.16*	-.26*	-.19*	-.36*	-.24*	.70*	-	
18. STAI-T (W2)	916	47.38	8.47	-.32*	.09	-.13*	-.03	-.26*	-.12*	.62*	.61*	.67*	-.31*	.14*	-.19*	-.15*	-.33*	-.22*	.73*	.82*	-

BIS, Behavioral Inhibition System; BAS, Behavioral Activation System; DARS-H, Anhedonia of Pastime/Hobbies; DARS-FD, Anhedonia of Food/Drink; DARS-SA, Anhedonia of Social Activities; DARS-SE, Anhedonia of Sensory Experiences; CDI, Child Depression Inventory; STAI-S, State Anxiety; STAI-T Trait Anxiety; W1, Wave 1; W2, Wave 2. *Indicates a significant correlation at the p <.000327 level based on Bonferroni adjustment for multiple comparisons.

T-test between two sexes was preformed to identify if any sex differences existed among main variables. In two waves of data, male participants were significantly higher than female participants in the BIS (higher level indicated a lower level of behavioral inhabitation), social activities subscale of DARS (higher score indicated a lower level of anhedonia), and wave 2 BAS (higher level indicated a lower level of behavioral activation). Female participants showed a greater level of depressive and anxiety symptoms level cross-sectionally and longitudinally (See **Supplementary Table S2**).

### 3.2 Mediating effects of multiple domains of anhedonia

**Figure 1:**
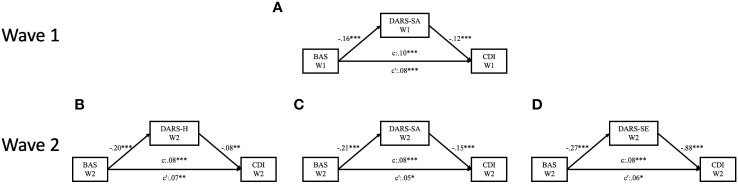
Diagrams for the paths of the cross-sectionally mediating effect of anhedonia on the relationships between behavioral activation and depressive symptoms, with state anxiety and trait anxiety levels controlled. **(A)** Mediating effect of social activities domain of anhedonia at baseline; **(B)** Mediating effect of social activities domain of anhedonia three months later; **(C)** Mediating effect of hobbies domain of anhedonia three months later; **(D)** Mediating effect of sensory experiences domain of anhedonia three months later. *p <.05; **p <.01; ***p <.001.

Mediation analysis was performed to examine the indirect effects of the subscales of DARS on the relationship between BAS and CDI scores. As shown in **Figure 1**, four models were used, controlling for the scores of the STAI-T and the STAI-S. During Wave 1, it was observed that the social activities domain of anhedonia served as a mediator between BAS and CDI scores (See Model A in **Figure 1**). The mediating effects of the social activities domain of anhedonia accounted for 22.33% of the total effect (See Model A in **Supplementary Table S1**). In the follow-up data, it was observed that the domains of social activities, hobbies, and sensory experiences mediated the relationship between BAS and CDI scores (See Model B to Model D in **Figure 1**). The mediating effects of these domains accounted for 37.80%, 18.29%, and 31.71%, respectively, of the total effect (See Model B to Model D in **Supplementary Table S1**). Furthermore, significant direct effects between BAS and depression were consistently observed across the two waves of the survey, indicating a robust relationship (See Model A to Model H in **Supplementary Table S1**).

**Figure 2:**
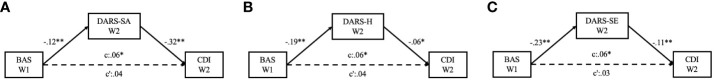
Diagrams for the paths of the longitudinally mediating effect of anhedonia on the relationships between behavioral activation and depressive symptoms, with state anxiety and trait anxiety levels controlled. **(A)** Longitudinal mediating effect of social activities domain of anhedonia; **(B)** Longitudinal mediating effect of hobbies domain of anhedonia; **(C)** Longitudinal mediating effect of sensory experiences domain of anhedonia. *p <.05; **p <.01.

The findings of the longitudinal mediation tests revealed that the domains of social activities in anhedonia at Wave 2 directly mediated the longitudinal relationship between BAS scores at Wave 1 and CDI scores at Wave 2 while controlling for STAI-S and STAI-T levels at Wave 2 (See Model A in **Figure 2**). Additionally, similar mediating effect were observed for the domains of hobbies (See Model B in **Figure 2**) and sensory experiences (See Model C in **Figure 2**). The mediating effects of the social activities, hobbies, and sensory experiences domains of anhedonia accounted for 51.79%, 21.43%, and 42.86%, respectively, of the total effect as demonstrated in three models (see in Model I to Model L in **Supplementary Table S1**).

**Figure 3:**
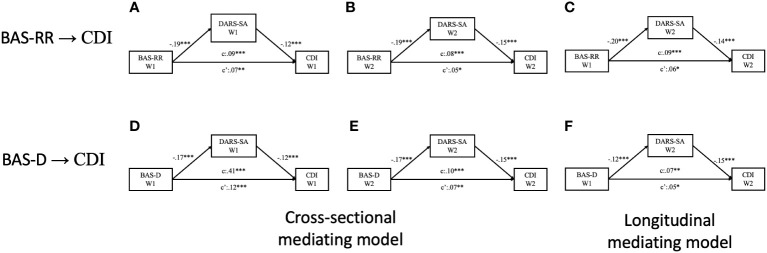
Diagrams for the paths of the mediating effect of social activities domain of anhedonia between subscales of behavioral activation and depressive symptoms, with state anxiety and trait anxiety levels controlled. Model A to Model C are mediating models of reward-responsiveness subscale of behavioral activation predicting depressive symptoms; Model D to Model F are mediating models of drive subscale of behavioral activation predicting depressive symptoms. **(A)** Cross-sectional mediating effect of social activities domain of anhedonia based on wave 1 data; **(B)** Cross-sectional mediating effect of social activities domain of anhedonia based on wave 2 data; **(C)** Longitudinal mediating effect of social activities domain of anhedonia; **(D)** Cross-sectional mediating effect of social activities domain of anhedonia based on wave 1 data; **(E)** Cross-sectional mediating effect of social activities domain of anhedonia based on wave 2 data; **(F)** Longitudinal mediating effect of social activities domain of anhedonia. *p <.05; **p <.01; ***p <.001.

To further investigate how different dimensions of BAS had effect on the level of depressive symptoms through anhedonia domains, a subsequent mediating effect analysis was performed. Specifically, the mediation models examined the impact of the four domains of anhedonia on depressive symptoms while considering the scores in the three subscales of BAS (i.e., reward responsiveness, drive, and fun-seeking). The results revealed that the social activities, hobbies, sensory experiences domains of anhedonia significantly mediated the effects in between both models that reward responsiveness, and drive subscale of BAS predicting scores on the CDI cross-sectionally (See Model A, B, D, E in **Figure 3**; **Supplementary Figure S1**) and longitudinally (See Model C, F in **Figure 3**; **Supplementary Figure S2**). Nevertheless, we did not observe any direct and indirect effect on the relationship between fun-seeking dimension and the level of depressive symptoms.

Considering that anhedonia is a significant symptom of depression, we excluded the items related to anhedonia from the total score of CDI and performed additional mediation modeling. Remarkably, the outcomes of these analyses were consistent with those in the main models, reaffirming the findings. Results were illustrated in **Supplementary Materials** (see **Supplementary Table S2**).

### 3.3 Subdomain comparison analysis regarding anhedonia in the social activities

Additionally, Wald tests were conducted to assess whether the proportions of beta-values representing the mediating effects differed among the four domains of anhedonia in various models. It was observed that the social activities domain exhibited significantly higher values than hobbies, *W (1*) = 5.046, *p* <.05, and foods/drinks, *W (1*) = 19.86, *p* <.001, at wave 2 data. A similar pattern of results emerged in the longitudinal model (Social activities vs. hobbies: *W (1*) = 5.227, *p* <.05; social activities vs. food/drinks: *W (1*) = 18.197, *p* <.001). Nevertheless, no significant differences were noted between the social activities and sensory experiences domains of anhedonia in both cross-sectional and longitudinal models. Given that the social activities domain was the sole significant mediator between BAS and depressive symptoms, no Wald test was conducted in the models using Wave 1 data.

Upon further examination of the mediating effects between BAS sub-components and depressive symptoms, it became evident that Reward Responsiveness had a significant impact on level of depressive symptoms through the social activities domain of anhedonia, which was notably higher than the effects observed in the food/drinks and hobbies domains in the cross-sectional data (Social activities vs. hobbies: *W (1*) = 3.863, *p* <.05; social activities vs. food/drinks: *W (1*) = 15.908, *p* <.001) and longitudinal dataset (Social activities vs. hobbies: *W (1*) = 6.044, *p* <.05; social activities vs. food/drinks: *W (1*) = 12.468, *p* <.001). Similar findings were observed, indicating that anhedonia within the social activity domain had a more substantial impact on the association between the drive component of BAS and the level of depressive symptoms compared to the food/drinks domain of anhedonia, both in the cross-sectional, *W*(1) = 16.896, *p* <.001 and longitudinal dataset, *W*(1) = 17.678, *p* <.001.

Finally, in view of CDI containing anhedonia items, further Wald test results were carried out to determine the mediating effect of anhedonia domains on the relationships between BAS subdomain and level of depressive symptoms score excluded anhedonia subscale cross-sectionally (Social activities vs. hobbies: *W*(1) = 3.912, *p* <.05; social activities vs. food/drinks: *W*(1) = 16.129, *p* <.001) and longitudinally (Social activities vs. hobbies: *W*(1) = 9.362, *p* <.05; social activities vs. food/drinks: *W*(1) = 17.497, *p* <.001).

### 3.4 Moderating effect of sex

Finally, the moderating effects of sex were examined in the mediating models. However, as shown in **Tables 2**, **3**, sex was not a significant moderator in the cross-sectional and longitudinal relationships between BAS and depressive symptoms.

**Table 2:** Moderating effects of sex on the cross-sectional relationship between behavioral activation, anhedonia and depressive symptoms.

		β	95% CI	β	95% CI
		Wave 1		Wave 2	
**Sex (1=male;2=female)**	**Model A(W1)/Model E(W2)**				
	**Direct path**				
	BAS × Sex → Depressive symptoms	0.005	[-0.253, 0.265]	-0.009	[-0.219, 0.194]
	**Indirect path**				
	BAS × Sex → DARS-H	0	[-0.327, 0.341]	0.273	[-0.100, 0.642]
	DARS-H × Sex → Depressive symptoms	0.005	[-0.253, 0.265]	0.009	[-0.253, 0.255]
	**Model B(W1)/Model F(W2)**				
	**Direct path**				
	BAS × Sex → Depressive symptoms	-0.062	[-0.311, 0.182]	-0.083	[-0.292, 0.121]
	**Indirect path**				
	BAS × Sex → DARS-SA	-0.169	[-0.516, 0.181]	-0.095	[-0.463, 0.310]
	DARS-SA × Sex → Depressive symptoms	-0.010	[-0.247, 0.218]	-0.140	[-0.341, 0.069]
	**Model C(W1)/Model G(W2)**				
	**Direct path**				
	BAS × Sex → Depressive symptoms	-0.049	[-0.299, 0.206]	-0.036	[-0.251, 0.172]
	**Indirect path**				
	BAS × Sex → DARS-SE	-0.210	[-0.575, 0.127]	0.162	[-0.218, 0.553]
	DARS-SE × Sex → Depressive symptoms	-0.031	[-0.255, 0.198]	-0.071	[-0.301, 0.170]
	**Model D(W1)/Model H(W2)**				
	**Direct path**				
	BAS× Sex → Depressive symptoms	0.113	[-0.054, 0.285]	-0.030	[-0.246, 0.179]
	**Indirect path**				
	BAS × Sex → DARS-FD	-0.181	[-0.568, 0.202]	0.055	[-0.308, 0.453]
	DARS-FD × Sex → Depressive symptoms	0.097	[-0.115, 0.298]	0.003	[-0.222, 0.216]

BIS, Behavioral Inhibition System; BAS, Behavioral Activation System; DARS-H, Anhedonia of Pastime/Hobbies; DARS-FD, Anhedonia of Food/Drink; DARS-SA, Anhedonia of Social Activities; DARS-SE, Anhedonia of Sensory Experiences; The moderating effects of sex were examined based on the Model A to H. Model A to D was set up to determine the mediating effect of four anhedonia dimensions based on wave 1 data; Model E to H was set up to determine the mediating effect of four anhedonia dimensions based on wave 2 data.

**Table 3:** Moderating effects of sex on the longitudinal relationship between behavioral activation, anhedonia and depressive symptoms.

		β	95% CI
**Sex (1=male;2=female)**	**Model I**		
	**Direct path**		
	BAS × Sex → Depressive symptoms	0.023	[-0.263, 0.337]
	**Indirect path**		
	BAS × Sex → DARS-H	0.242	[-0.112, 0.620]
	DARS-H × Sex → Depressive symptoms	-0.178	[-0.489, 0.170]
	**Model J**		
	**Direct path**		
	BAS × Sex → Depressive symptoms	0.031	[-0.282, 0.369]
	**Indirect path**		
	BAS × Sex → DARS-SA	-0.208	[-0.537, 0.125]
	DARS-SA × Sex → Depressive symptoms	-0.085	[-0.343, 0.193]
	**Model K**		
	**Direct path**		
	BAS × Sex → Depressive symptoms	0.075	[-0.242, 0.415]
	**Indirect path**		
	BAS × Sex → DARS-SE	-0.043	[-0.377, 0.303]
	DARS-SE × Sex → Depressive symptoms	0.030	[-0.273, 0.338]
	**Model L**		
	**Direct path**		
	BAS × Sex → Depressive symptoms	0.086	[-0.218, 0.422]
	**Indirect path**		
	BAS × Sex → DARS-FD	-0.115	[-0.449, 0.224]
	DARS-FD × Sex → Depressive symptoms	0.062	[-0.193, 0.320]

BIS, Behavioral Inhibition System; BAS, Behavioral Activation System; DARS-H, Anhedonia of Pastime/Hobbies; DARS-FD, Anhedonia of Food/Drink; DARS-SA, Anhedonia of Social Activities; DARS-SE, Anhedonia of Sensory Experiences; The moderating effects of sex were examined based on the Model I to L; Model I to L was built up to examine the longitudinal mediating effect of four anhedonia dimensions at wave 2 between BAS at wave 1, and depressive symptoms at wave2.

## 4 Discussion

This study investigated the mediating role of anhedonia in the relationship between BAS and depressive symptoms among adolescents, both cross-sectionally and longitudinally. As hypothesized, the social activities domain of anhedonia consistently demonstrated a significant indirect effect on the association between BAS and depressive symptoms across two waves of data. Notably, the hedonic capacity within the realm of social activities emerged as the foremost mediator in elucidating the connection between BAS and depressive symptoms. The outstanding mediating role of social activities domain of anhedonia remained significant on the relationship between Reward Responsiveness and Drive dimensions of BAS, predicting level of depressive symptoms cross-sectionally and longitudinally. Additionally, no significant moderation by sex was found in relation to BAS, anhedonia, and depressive symptoms.

### 4.1 Mediating effect of the different anhedonia domains

In line with the proposed hypothesis, BAS levels could predict a reduction in depressive symptoms, where anhedonia plays a significant mediating role in this relationship. This finding has potential implications for treating depressive symptoms, particularly in the context of BAT. The core mechanism of BAT involves increasing approach motivation (8) and reducing avoidance behavior (9–11), both of which are closely related to BAS. A decrease in anhedonia levels has been proposed as the underlying mechanism behind the effectiveness of BAT (8, 38). Studies applying BAT to individuals with depression have shown longitudinal improvements in anhedonia symptoms (38, 39). Thus, this study provides empirical evidence supporting the effectiveness of BAT and supports its underlying mechanism in treating depression by enhancing motivation toward rewards.

Furthermore, the mediating effect of the social activities domain of anhedonia was consistently robust in the relationship between BAS and depressive symptoms across different time points. This finding aligns with a previous study that demonstrated the significance of social anhedonia in predicting depression longitudinally among adolescents (20). Deficits in social functioning, including social anhedonia, have been recognized as early signs of impending depression-related problems (40). Adolescence is a critical period for the development of social cognition and learning, such as emotion regulation (41), facial recognition, and empathy (42). These functions play a vital role in facilitating the development of adolescents, enabling them to experience more pleasant feelings during social interactions and motivating them to engage in various social activities (43, 44).

Studies investigating social learning in early adolescence have revealed an increased preference for majority opinions (e.g., liked by everyone) compared with that in childhood (45). The results of this study further support the importance of social learning in typical adolescent development by identifying a potential link between decreased approach motivation and reduced engagement with social stimuli, both cross-sectionally and longitudinally. This decreased frequency of approaching favorable social stimuli could potentially contribute to an increased frequency of negative emotions, thereby enhancing the risk of developing depression (46).

Taken together, it is reasonable to believe that anhedonia, particularly within social activities domain, plays a critical role in mediating the relationship between BAS and depressive symptoms in adolescents. To the best of our knowledge, this is the first study to compare models of the longitudinal mediating effect from different domains of anhedonia between BAS and depressive symptoms.

### 4.2 Different role of BAS dimensions

We found a significant mediating effect of anhedonia in the social activities on the relationship between Reward Responsiveness and Drive dimensions of BAS, which predicts depressive symptoms cross-sectionally and longitudinally. Consistent with our findings, prior research has already unveiled the predictive nature of the drive and reward-responsiveness subscales in determining participants’ responses to reward and their experience of pleasure over time (28). One previous study proposed that apathy and anhedonia are two distinct symptoms that may share mechanisms related to effort-based decision making (47). Apathy is characterized as a state where individuals experience a decrease or loss of motivation (48). Accordingly, the BAS-Drive subscale is highly related to apathy as it composes items that measuring motivation towards reward and achievement. On the other hand, anhedonia refers to a persistent and notable decline in the interest and pleasure of activities (47). The BAS-reward responsiveness subscale estimates the experiences of excitement and positive emotion after participants received rewards. It is evident that anhedonia is closely related to BAS-reward responsiveness. Additionally, it is noteworthy that the mediating effect did not exert an influence on the Fun Seeking component of BAS or the level of depressive symptoms significantly. One possible explanation is that unlike the Reward Responsiveness and Drive components, the Fun Seeking component primarily relates to the inclination to engage in potentially rewarding situations without the need for extensive effort-based decision-making, which might not strongly contribute to the development of depression (49). Hence, our findings suggest that anhedonia within the social activities domain could play a significant role in shaping the relationships between depressive symptoms and various motivation components that involve more effort expenditure.

### 4.3 Moderating effect of sex

Unlike previous evidence highlighting the moderating role of sex in the relationship between predictors and anhedonia (50), as well as depression (51), the results of this study did not identify any significant moderating effect of sex on the relationship between BAS, anhedonia, and depressive symptoms. One possible explanation for this discrepancy, specifically regarding anhedonia, could be attributed to the development of the DARS (19), which aimed to mitigate sex bias. The *t*-test results comparing DARS scores between males and females only revealed a significant difference in social anhedonia. Additionally, both males and females reported similar preferences for hobbies (e.g., watching short videos), food/drink (e.g., fried foods), and sensory experiences (e.g., listening to music). Moreover, the DARS measured the participants’ state of anhedonia (19), and studies focusing on adolescent state of anhedonia did not report significant sex differences (20, 52).

Furthermore, studies investigating the treatment of adolescent depression have failed to establish sex as a significant moderator between cognitive factors (e.g., cognitive distortions and maladaptive beliefs, cognitive avoidance, and solution-focused thinking) and depression (53). This suggests that sex does not play a prominent role in the relationship between depression and factors, such as BAS (28, 54) or anhedonia.

### 4.4 Limitations and future direction

The current study is subject to several limitations. Firstly, none of the participants underwent mental health evaluations by psychiatrists. Instead, their depressive levels were assessed solely using self-rating scales, which limited our ability to determine whether participants provide reliable evaluation on their level of depressive symptoms. To explore the complex interplay between BAS, anhedonia, and depression more comprehensively, future researchers should consider integrating both clinical such as Hamilton Depression Scale and self-report assessments. Secondly, it is important to note that all participants in this study were typically-developing adolescents. As a result, the reported levels of anhedonia and depressive symptoms may not be directly comparable to clinical levels. In the future, exploring these relationships in clinical samples, such as major depressive disorders, may yield more significant findings, particularly regarding moderating effect of sex on the relationship between BAS and depressive symptoms.

## 5 Conclusions

In summary, our findings provide supports for the mediating role of anhedonia, particularly within the social activities domain, in the relationship between BAS and depressive symptoms. These findings contribute to a deeper understanding of the mechanisms underlying the development of depressive symptoms and hold implications for developing innovative treatment approaches.

## Data availability statement

The datasets presented in this article are not readily available because No dataset available. Requests to access the datasets should be directed to Chao Yan, cyan@psy.ecnu.edu.cn.

## Ethics statement

The studies involving humans were approved by Ethics Committee on Human Research of East China Normal University. The studies were conducted in accordance with the local legislation and institutional requirements. Written informed consent for participation in this study was provided by the participants’ legal guardians/next of kin.

## Author contributions

C-SK: Formal analysis, Methodology, Visualization, Writing – original draft. Q-YL: Formal analysis, Funding acquisition, Visualization, Writing – original draft. G-MX: Methodology, Writing – review & editing. H-YZ: Supervision, Writing – review & editing. J-HN: Funding acquisition, Writing – review & editing. CY: Conceptualization, Funding acquisition, Supervision, Writing – review & editing.
