# Supplementary material for: Diminished hedonic capacity in social activities as a mediator of the link between dysfunctional behavioral activation system and depressive symptoms
Source: Front Psychiatry. 2024 Feb 6;15:1337847. doi: 10.3389/fpsyt.2024.1337847 (PMC10876889; doi:10.3389/fpsyt.2024.1337847)
Supplement: Supplementary file 1 [file DataSheet_1.docx]

**SUPPLEMENTARY MATERIAL:**


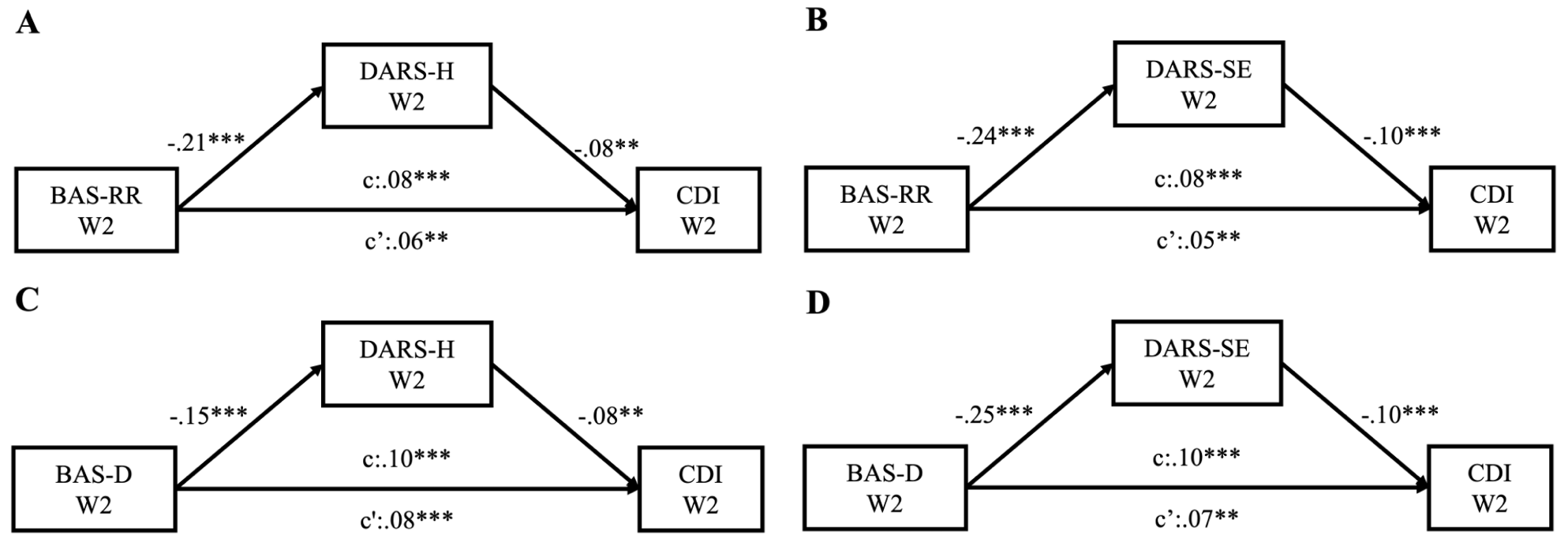


**Figure 1S: Cross-sectional mediating model between reward-responsiveness subscale of behavioral activation and depressive symptoms.**

A: Cross-sectional mediating model of hobbies domain of anhedonia between reward-responsiveness subscale of behavioral activation and depressive symptoms at wave 2. B: Cross-sectional mediating model of sensory experiences domain of anhedonia between reward-responsiveness subscale of behavioral activation and depressive symptoms at wave 2. C: Longitudinal mediating model of hobbies domain of anhedonia between reward-responsiveness subscale of behavioral activation and depressive symptoms at wave 2. D: Longitudinal mediating model of sensory experiences domain of anhedonia between reward-responsiveness subscale of behavioral activation and depressive symptoms at wave 2. Notes. ***p* < .01; ****p* < .001.


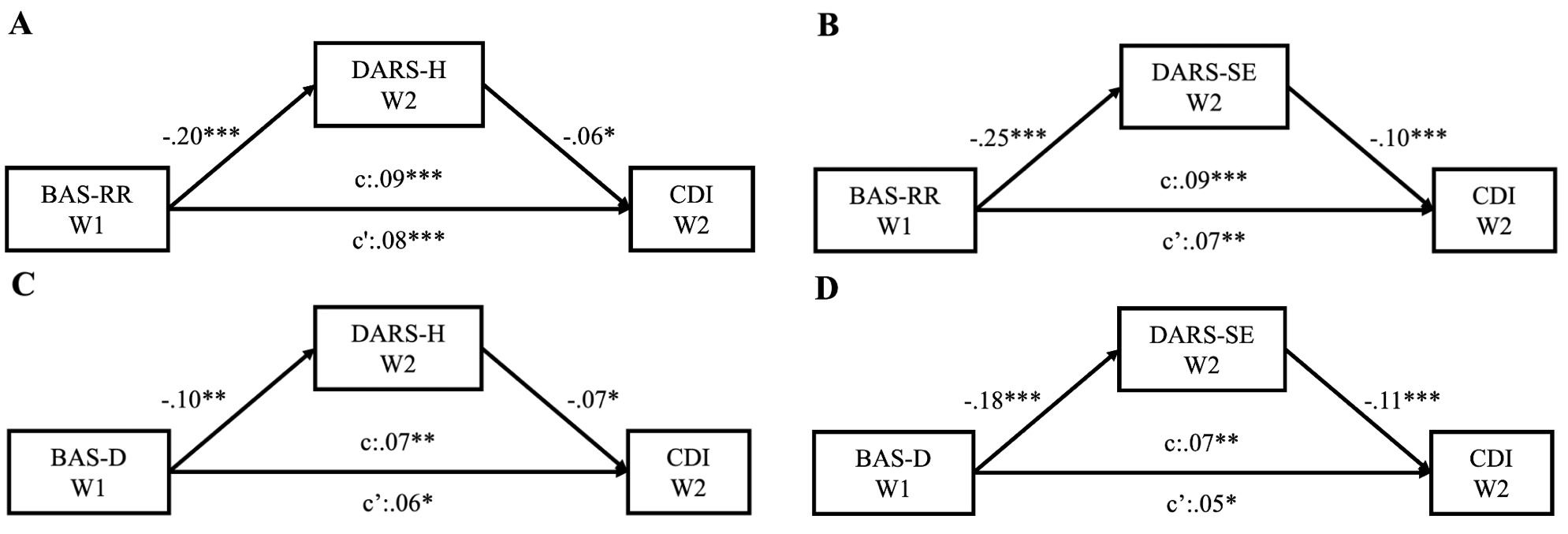


**Figure 2S: Longitudinal mediating model between drive subscale of behavioral activation and depressive symptoms.**

A: Cross-sectional mediating model of hobbies domain of anhedonia between drive subscale of behavioral activation and depressive symptoms at wave 2. B: Cross-sectional mediating model of sensory experiences domain of anhedonia between drive subscale of behavioral activation and depressive symptoms at wave 2. C: Longitudinal mediating model of hobbies domain of anhedonia between drive subscale of behavioral activation and depressive symptoms at wave 2. D: Longitudinal mediating model of sensory experiences domain of anhedonia between drive subscale of behavioral activation and depressive symptoms at wave 2. Notes. **p* < .05; ***p* < .01; ****p* < .001.

| **Table 1S** Mediating effects of anhedonia between behavioral activation and depressive symptoms | | | | | | | | | | |
| --- | --- | --- | --- | --- | --- | --- | --- | --- | --- | --- |
|  | **Cross-sectional data analysis** | | | | |  | **Longitudinal data analysis** | | | |
|  | **Wave 1** | | | **Wave 2** | |  | **Wave 1 (BAS) 🡪 Wave 2 (Depressive Symptoms)** | | | |
|  | **β** | **95% CI** | **β** | | **95% CI** |  | | **β** | | **95% CI** |
| **Total effect** |  |  |  | |  |  | |  | |  |
| BAS → Depressive symptoms | 0.103 | [0.057, 0.152] | 0.082 | | [0.038, 0.128] |  | | 0.056 | | [0.007, 0.104] |
|  |  |  |  | |  |  | |  | |  |
|  | **Model A** |  | **Model E** | |  |  | | **Model I** | |  |
| **Direct effect** |  |  |  | |  |  | |  | |  |
| BAS → Depressive symptoms | 0.105 | [0.057, 0.154] | 0.067 | | [0.023, 0.112] |  | | 0.044 | | [-0.007, 0.092] |
| **Indirect effect** |  |  |  | |  |  | |  | |  |
| BAS → DARS-H → Depressive symptoms | -0.001 | [-0.010, 0.008] | 0.015 | | [0.006, 0.030] |  | | 0.012 | | [0.003, 0.022] |
|  |  |  |  | |  |  | |  | |  |
|  | **Model B** |  | **Model F** | |  |  | | **Model J** | |  |
| **Direct effect** |  |  |  | |  |  | |  | |  |
| BAS → Depressive symptoms | 0.080 | [0.022, 0.130] | 0.052 | | [0.008, 0.095] |  | | 0.028 | | [-0.025, 0.078] |
| **Indirect effect** |  |  |  | |  |  | |  | |  |
| BAS → DARS-SA → Depressive symptoms | 0.023 | [0.012, 0.039] | 0.031 | | [0.017, 0.049] |  | | 0.029 | | [0.016, 0.045] |
|  |  |  |  | |  |  | |  | |  |
|  | **Model C** |  | **Model G** | |  |  | | **Model K** | |  |
| **Direct effect** |  |  |  | |  |  | |  | |  |
| BAS → Depressive symptoms | 0.101 | [0.053, 0.152] | 0.056 | | [0.011, 0.102] |  | | 0.032 | | [-0.021, 0.082] |
| **Indirect effect** |  |  |  | |  |  | |  | |  |
| BAS → DARS-SE → Depressive symptoms | 0.004 | [-0.009, 0.014] | 0.026 | | [0.013, 0.044] |  | | 0.024 | | [0.012, 0.040] |
|  |  |  |  | |  |  | |  | |  |
|  | **Model D** |  | **Model H** | |  |  | | **Model L** | |  |
| **Direct effect** |  |  |  | |  |  | |  | |  |
| BAS → Depressive symptoms | 0.104 | [0.055, 0.154] | 0.077 | | [0.031, 0.124] |  | | 0.052 | | [0.000, 0.102] |
| **Indirect effect** |  |  | |  |  |  |  | |  | |
| BAS → DARS-FD → Depressive symptoms | -0.001 | [-0.011, 0.010] | 0.005 | | [-0.005, 0.017] |  | | 0.005 | | [-0.004, 0.015] |

***Notes.*** BIS: Behavioral Inhibition System; BAS: Behavioral Activation System; DARS-H: Pastime/Hobbies Domain of Anhedonia; DARS-FD: Food/Drink Domain of Anhedonia; DARS-SA: Social Activities Domain of Anhedonia; DARS-SE: Sensory Experiences Domain of Anhedonia; Model A to D was set up to determine the mediating effect of four anhedonia dimensions at baseline; Model E to H was set up to determine the mediating effect of four anhedonia dimensions at three months later; Model I to L was built up to examine the longitudinal mediating effect of four anhedonia dimensions.

**Table 2S** Result of T-test analysis examining the sex difference of main variables in two waves of survey

|  | **Male** | | **Female** | | | **df** | ***t*** | ***p*** | **Cohen’s d** |  |
| --- | --- | --- | --- | --- | --- | --- | --- | --- | --- | --- |
|  | **M** | **SD** | | **M** | **SD** |  |  |  |  |  |
| **Wave1** | | | | | | | | | |  |
| BIS-W1 | 8.88 | 2.959 | | 7.94 | 2.616 | 758.290 | 5.204*** | <.001 | 0.341 |  |
| BAS-W1 | 24.62 | 6.683 | | 24.50 | 5.659 | 732.790 | 0.313 | .755 | 0.02 |  |
| DARS-H-W1 | 13.14 | 3.129 | | 13.17 | 3.030 | 1021 | -0.160 | .873 | 0.01 |  |
| DARS-FD-W1 | 10.86 | 3.574 | | 11.24 | 3.191 | 764.197 | -1.729 | .084 | 0.114 |  |
| DARS-SA-W1 | 12.27 | 3.448 | | 11.77 | 3.495 | 1021 | 2.225* | .026 | 0.144 |  |
| DARS-SE-W1 | 15.77 | 3.983 | | 15.48 | 4.134 | 1021 | 1.122 | .262 | 0.071 |  |
| CDI-W1 | 15.03 | 7.339 | | 16.84 | 7.431 | 1021 | -3.814*** | .000 | 0.245 |  |
| STAIS-W1 | 44.06 | 10.804 | | 45.44 | 11.029 | 1021 | -1.961* | .050 | 0.126 |  |
| STAIT-W1 | 46.25 | 8.509 | | 48.04 | 8.581 | 1021 | -3.265*** | .001 | 0.209 |  |
|  | | | | | | | | | |  |
| **Wave2** | | | | | | | | | |  |
| BIS-W2 | 9.45 | 3.246 | | 8.11 | 2.808 | 645.747 | 6.341*** | <.001 | 0.45 |  |
| BAS-W2 | 25.53 | 7.294 | | 24.48 | 6.093 | 627.898 | 2.245* | .025 | 0.16 |  |
| DARS-H-W2 | 13.02 | 3.457 | | 13.06 | 3.234 | 914 | -0.158 | .875 | 0.012 |  |
| DARS-FD-W2 | 11.10 | 3.487 | | 11.26 | 3.238 | 914 | -0.691 | .490 | 0.048 |  |
| DARS-SA-W2 | 12.22 | 3.586 | | 11.65 | 3.684 | 914 | 2.289* | .022 | 0.156 |  |
| DARS-SE-W2 | 15.42 | 4.219 | | 15.44 | 4.153 | 914 | -0.068 | .946 | 0.005 |  |
| CDI-W2 | 15.49 | 7.995 | | 17.01 | 7.661 | 914 | -2.858** | .004 | 0.195 |  |
| STAIS-W2 | 44.17 | 11.085 | | 46.05 | 10.951 | 914 | -2.502* | .013 | 0.171 |  |
| STAIT-W2 | 46.28 | 8.815 | | 48.05 | 8.184 | 914 | -3.078** | .002 | 0.21 |  |

***Notes.*** *p < .05, **p < .01, ***p < .001*;* SD: Standard Deviation; BIS: Behavioral Inhabitation System; BAS: Behavioral Activation System; DARS-H: DARS Hobbies Subscale; DARS-B: DARS Food and Drink Subscale; DARS-C: DARS Social Activities Subscale; DARS-D: DARS Sensory Experience Subscale; CDI: Children’s Depression Inventory; STAIS: State Anxiety; STAIT: Trait Anxiety. Mean parameter values for each of the analyses are shown for the wave 1 male (n = 394), female (n = 629), and wave 2 male (n = 345) and female (n = 571) as well as the results of t tests comparing the parameter estimates between the two ages.

| **Table 3S** Mediating effects of anhedonia between behavioral activation and depressive symptoms excluded anhedonia subscale | | | | | | | |
| --- | --- | --- | --- | --- | --- | --- | --- |
|  | **Cross-sectional data analysis** | | | |  | **Longitudinal data analysis** | |
|  | **Wave 1** | | **Wave 2** | |  | **Wave 1 (BAS) 🡪 Wave 2 (CDI-4)** | |
|  | **β** | **95% CI** | **β** | **95% CI** |  | **β** | **95% CI** |
| **Total effect** |  |  |  |  |  |  |  |
| BAS → CDI-4 | 0.084 | [0.036, 0.135] | 0.062 | [0.014, 0.112] |  | 0.070 | [0.014, 0.127] |
|  |  |  |  |  |  |  |  |
|  | **Model A** |  | **Model E** |  |  | **Model I** |  |
| **Direct effect** |  |  |  |  |  |  |  |
| BAS → CDI-4 | 0.090 | [0.039, 0.142] | 0.050 | [0.002, 0.098] |  | 0.059 | [0.001, 0.121] |
| **Indirect effect** |  |  |  |  |  |  |  |
| BAS → DARS-H → CDI-4 | -0.006 | [-0.016, 0.003] | 0.012 | [0.003, 0.026] |  | 0.011 | [0.002, 0.025] |
|  |  |  |  |  |  |  |  |
|  | **Model B** |  | **Model F** |  |  | **Model J** |  |
| **Direct effect** |  |  |  |  |  |  |  |
| BAS → CDI-4 | 0.064 | [0.015, 0.118] | 0.037 | [-0.012, 0.088] |  | 0.064 | [0.007, 0.120] |
| **Indirect effect** |  |  |  |  |  |  |  |
| BAS → DARS-SA → CDI-4 | 0.019 | [0.009, 0.034] | 0.025 | [0.013, 0.042] |  | 0.006 | [-0.004, 0.018] |
|  |  |  |  |  |  |  |  |
|  | **Model C** |  | **Model G** |  |  | **Model K** |  |
| **Direct effect** |  |  |  |  |  |  |  |
| BAS → CDI-4 | 0.085 | [0.042, 0.139] | 0.043 | [-0.008, 0.095] |  | 0.042 | [-0.016, 0.100] |
| **Indirect effect** |  |  |  |  |  |  |  |
| BAS → DARS-SE → CDI-4 | -0.001 | [-0.013, 0.011] | 0.019 | [0.006, 0.035] |  | 0.028 | [0.016, 0.045] |
|  |  |  |  |  |  |  |  |
|  | **Model D** |  | **Model H** |  |  | **Model L** |  |
| **Direct effect** |  |  |  |  |  |  |  |
| BAS → CDI-4 | 0.086 | [0.035, 0.139] | 0.058 | [0.007, 0.111] |  | 0.047 | [-0.010, 0.105] |
| **Indirect effect** |  |  |  |  |  |  |  |
| BAS → DARS-FD → CDI-4 | -0.002 | [-0.013, 0.009] | 0.004 | [-0.008, 0.016] |  | 0.023 | [0.010, 0.039] |

***Notes.*** BIS: Behavioral Inhibition System; BAS: Behavioral Activation System; DARS-H: Pastime/Hobbies Domain of Anhedonia; DARS-FD: Food/Drink Domain of Anhedonia; DARS-SA: Social Activities Domain of Anhedonia; DARS-SE: Sensory Experiences Domain of Anhedonia; CDI-4: Score of Child Depression Inventory Excluded Anhedonia Subscale; Model A to D was set up to determine the mediating effect of four anhedonia dimensions at baseline; Model E to H was set up to determine the mediating effect of four anhedonia dimensions at three months later; Model I to L was built up to examine the longitudinal mediating effect of four anhedonia dimensions.
